# A Robust Metabolic Enzyme-Based Prognostic Signature for Head and Neck Squamous Cell Carcinoma

**DOI:** 10.3389/fonc.2021.770241

**Published:** 2022-01-20

**Authors:** Zizhao Mai, Huan Chen, Mingshu Huang, Xinyuan Zhao, Li Cui

**Affiliations:** ^1^ Stomatological Hospital, Southern Medical University, Guangzhou, China; ^2^ Division of Oral Biology and Medicine, School of Dentistry, University of California, Los Angeles, CA, United States

**Keywords:** head and neck squamous cell carcinoma (HNSCC), metabolic enzyme, prognostic signature, survival analysis, The Cancer Genome Atlas

## Abstract

**Background:**

Head and neck squamous cell carcinoma (HNSCC) is still a menace to public wellbeing globally. However, the underlying molecular events influencing the carcinogenesis and prognosis of HNSCC are poorly known.

**Methods:**

Gene expression profiles of The Cancer Genome Atlas (TCGA) HNSCC dataset and GSE37991 were downloaded from the TCGA database and gene expression omnibus, respectively. The common differentially expressed metabolic enzymes (DEMEs) between HNSCC tissues and normal controls were screened out. Then a DEME-based molecular signature and a clinically practical nomogram model were constructed and validated.

**Results:**

A total of 23 commonly upregulated and 9 commonly downregulated DEMEs were identified in TCGA HNSCC and GSE37991. Gene ontology analyses of the common DEMEs revealed that alpha-amino acid metabolic process, glycosyl compound metabolic process, and cellular amino acid metabolic process were enriched. Based on the TCGA HNSCC cohort, we have built up a robust DEME-based prognostic signature including *HPRT1*, *PLOD2*, *ASNS*, *TXNRD1*, *CYP27B1*, and *FUT6* for predicting the clinical outcome of HNSCC. Furthermore, this prognosis signature was successfully validated in another independent cohort GSE65858. Moreover, a potent prognostic signature-based nomogram model was constructed to provide personalized therapeutic guidance for treating HNSCC. *In vitro* experiment revealed that the knockdown of TXNRD1 suppressed malignant activities of HNSCC cells.

**Conclusion:**

Our study has successfully developed a robust DEME-based signature for predicting the prognosis of HNSCC. Moreover, the nomogram model might provide useful guidance for the precision treatment of HNSCC.

## Introduction

Head and neck squamous cell carcinoma (HNSCC) is cancer that arises from squamous cells in the area of the head and neck. HNSCC represents up to 90% of tumors in the head and neck region, which includes malignancy of the oral cavity, pharynx, and larynx (1, 2). The initiation and development of HNSCC are mainly caused by genetic alterations, human papillomavirus (HPV) infection, and consumption of tobacco, alcohol, and areca-nut, etc (3). Although surgery, radiochemotherapy, targeted therapy, and immunotherapy have been significantly advanced, patients with HNSCC have a median five-year overall survival (OS) rate of approximately 66% (4). HNSCC is usually treatable if detected at the earliest stage. Unfortunately, patients often present with advanced clinical stages at the time of diagnosis that is incurable or requires aggressive treatment, leading to an unfavorable prognosis (5). This highlights the significance of developing novel and robust molecular signatures for precisely evaluating the clinical outcome of HNSCC, which contributes to therapeutic guidance for HNSCC (6, 7).

Metabolic alterations of tumors were first found nearly a century ago, but only recently has reprogrammed metabolism been deemed as a cancer hallmark (8, 9). For example, nutrient uptake and biosynthesis are required in the early stages of cancer progression, while oxidative phosphorylation and oxidative stress resistance occur in the later stages of tumor growth (10). Therefore, reprogrammed metabolism has become a topic of renewed interest, and reversing abnormal metabolic processes might be a novel approach for the treatment of HNSCC (11). However, it is still uncertain which key metabolic enzymes affect the dismal prognosis of HNSCC.

The Cancer Genome Atlas (TCGA, http://cancergenome.nih.gov/) and NCBI-Gene Expression Omnibus (GEO, http://www.ncbi.nlm.nih.gov/geo/) are international public databases that archive and freely distribute next-generation sequencing, microarray, and other formats of high-throughput datasets, which are valuable resources for improving our understanding of cancer development (12, 13). In this study, we first identified the common differentially expressed metabolic enzymes (DEMEs) in TCGA HNSCC and GSE37991 cohort. Afterward a robust DEME-based signature was successfully built up and validated for predicting the clinical outcome of HNSCC. Moreover, a clinically practical nomogram model was constructed to accurately estimate HNSCC prognosis.

## Materials and Methods

### Data Source

The original GEO datasets GSE37991 and GSE65858 were downloaded from NCBI GEO databases. The data of GSE37991 and GSE65858 were based on GPL6883 (Illumina HumanRef-8 v3.0 expression beadchip) and GPL10558 (Illumina HumanHT-12 V4.0 expression beadchip), respectively. For the TCGA HNSCC cohort, the raw data and corresponding clinical information were downloaded from the TCGA data portal. The tumor located in lips, tongue, oral cavity, oropharynx, larynx, and hypopharynx were selected. The format of the TCGA HNSCC downloaded data was HTseq-FPKM, and the formats of GSE37991 and GSE65858 were normalized microarray data.

The RNA‐seq data of the TGCA HNSCC cohort included 521 HNSCC samples and 44 normal control samples. Forty‐three out of 44 normal samples were matched to the HNSCC tumor samples. Only one normal sample from the salivary gland was not matched to the tumor samples. Two hundred and seventy HNSCC tumor samples were included in the microarray data of GSE65858, which did not include normal control samples. The microarray data of the GSE37991 cohort included 40 HNSCC samples and 40 paired normal samples. Regarding the inclusion/exclusion criteria for the enrolled patients, both tumor and normal control samples in TCGA HNSCC and GSE37991 cohorts were included for the differential expression analysis. For the construction of risk signature, only the tumor samples in TCGA HNSCC, GSE37991 and GSE65858 cohorts were considered, and all normal control samples were excluded. The patients without clinical characteristics such as follow-up time, follow-up status, age, gender, clinical stage, T stage or N stage were excluded. Besides, the patients with the TX stage and NX stage were also excluded due to the disturbance to grouping. The clinical characteristics including age, gender, stage of the HNSCC tumor samples in the TCGA HNSCC discovery cohort and GSE65858 validation cohort were summarized in **Tables S1** and **S2**, respectively.

### Data Pre-Processing and Differential Expression Analysis

Briefly, the probes that have no expression in most of the samples were excluded. The FPKM data of the TCGA HNSCC cohort was converted to TPM format for further analysis. For GSE37991, data normalization was achieved with GeneSpring GX software (Agilent Technologies). For GSE65858, the data was first processed within the R/Bioconductor. Then the expression values were subjected to log_2_-transformation and the normalization was performed using Robust Spline Normalization (RSN). Batch effects of expression BeadChips were corrected using ComBat.

The DEMEs in GSE37991 and TCGA HNSCC cohort were identified by the edgeR package. Absolute log_2_FC>1 and *p*<0.05 were selected as the demarcation criteria based on Benjamini & Hochberg (BH) procedure. The common differentially expressed genes (DEGs) between GSE37991 and TCGA HNSCC cohort were identified by the intersect function in R.

### Gene Ontology Enrichment Analysis

Gene Ontology (GO) enrichment analysis was performed using the DAVID (the Database for Annotation, Visualization and Integration Discovery).

### Prognostic Signature Generation and Validation

TCGA HNSCC cohort and GSE65858 were used as discovery and validation cohorts, respectively. DEMEs were demonstrated to be correlative with the OS of HNSCC by univariate Cox proportional hazards regression analysis, which adopted the statistics from the TCGA HNSCC cohort. Subsequently, the most optimum DEMEs were chosen by the approach of the least absolute shrinkage and selection operator (LASSO) regression. The independent DEME-based prognostic signature was determined by the multivariate Cox proportional hazards regression analysis. Afterward a risk score model was constructed with this independent DEME-based prognostic signature. The summation of each DEME’s score was computed as a risk score for each HNSCC patient: $\text{risk score}=\Sigma_{i=1}^{n}\;\beta i\times Ei$ (14). On basis of the median value of the risk scores, the TCGA HNSCC cohort was divided into a low-risk group and a high-risk group. We compared the OS between low- and high-risk groups and assessed the differential survival distinguished by clinicopathological parameters between low- and high-risk groups. Similarly, the GSE65858 validation cohort was classified into low- and high-risk groups according to the above risk score model built up by the TCGA HNSCC cohort. Besides, the OS and the survival distinction were likewise differentiated by clinicopathological parameters between low- and high-risk groups.

### Nomogram Model Construction

A nomogram model including the risk score and other clinicopathological indices was constructed. The calibration curves were used to assess the accuracy for predicting 1-year OS and 3-year OS of HNSCC.

### Cell Culture and Transfection

Both SCC1 and SCC23 were cultured in Dulbecco’s modified Eagle’s medium (DMEM) (Gibco, USA) supplemented with 10% fetal bovine serum and 1% penicillin/streptomycin. Cells were grown in a 37°C, 5% CO_2_ cell incubator in a humidified atmosphere. The cells were transfected with siTXNRD1 and siCTRL using Lipofectamine^®^ RNA iMAX Transfection Reagent (Invitrogen, Carlsbad, CA, USA).

### Western Blot

The protein samples were separated on 4-20% SDS-PAGE gels and then transferred to polyvinylidene difluoride (PVDF) membranes. Following by blocking in 5% skimmed milk for 1 hr at room temperature, the blots were then probed with primary antibody against TXNRD1 (1:1000, Proteintech, Chicago, IL, USA) at 4°C overnight. The corresponding HRP-conjugated secondary antibody was used to incubate the membranes for 1 hr at room temperature. The signal was detected with ECL kits.

### MTT Assay

The cells were seeded into the 96‐wells of the plate at a density of 3,000 cells per well in 200 μl cell culture media. MTT solution (20 μl,5 mg/ml) was added to each well at the indicated time points and incubated for 4 hrs at 37°C. Dimethyl sulfoxide (DMSO) was added to dissolve the precipitate after removing the supernatant. A microculture plate reader (Tecan, Mannedorf, Switzerland) was used to measure the absorbance at 570 nm.

### 5‐Ethynyl‐2′‐Deoxyuridine Assay

According to the manufacturer’s instructions, the Click‐iT™ 5‐Ethynyl‐2′‐deoxyuridine (EdU) Cell Proliferation Kit for Imaging (Invitrogen) was used to perform the EdU assay. Briefly, EdU was added to the cells and incubated at 37°C for 2 hrs. Subsequently, 3.7% formaldehyde was used to fix the cells at room temperature for 20 min. After washing three changes of PBS, 0.5% Triton X‐100 was added to increase the permeability of the cellular membrane. 1× Click‐iT reaction cocktail was used to stain the cells in the dark at room temperature for 30 min. Then, the cell nucleus was stained by Hoechst 33342 dye. A confocal laser scanning microscope (Olympus, Center Valley, PA) was used to capture at least four random images per well.

### Statistical Analysis

The volcano plot and heatmaps were drawn by the “ggplot2” package of R software. The univariate and multivariate Cox regression analyses incorporated the clinical characteristics including age, gender, clinical stage, and risk score. The independent prognostic factors for HNSCC were identified by the univariate and multivariate Cox regression analyses. The Kaplan‐Meier method and log‐rank test were performed to calculate the OS distinguish between different groups. A *p*-value less than 0.05 is considered statistically significant.

## Results

### The Common DEMEs Between GSE37991 and TCGA HNSCC

**Figure 1:**
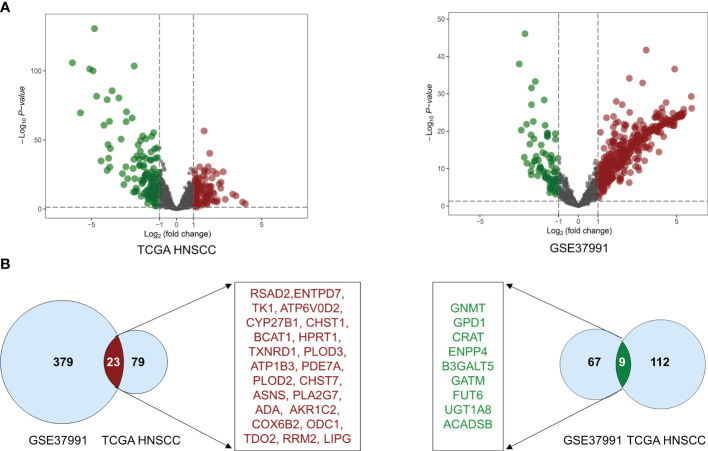
The significant alteration of DEMEs between TCGA HNSCC cohort and GSE37991. **(A)** The volcano plot showed significant alteration of DEMEs between normal controls and tumor tissues from HNSCC. The fold changes (log_2_‐scaled) were shown in the X-axis, and the *p* values (log_10_‐scaled) were shown in the Y-axis. Each gene was represented by a dot, and the significantly downregulated or upregulated DEMEs were represented by the green or red color. **(B)** The common DEMEs between the TCGA HNSCC cohort and GSE37991 were shown in Venn diagrams.

The volcano plot was used to visualize the distribution of metabolic enzymes between cancer and normal tissues from the GSE37991 and TCGA HNSCC cohort. The significantly downregulated or upregulated metabolic enzymes were represented as green or red dots, respectively (**Figure 1A**). In total, 478 (402 upregulated and 76 downregulated) and 223 (102 upregulated and 121 downregulated) significantly changed metabolic enzymes were identified in GSE37991 and TCGA HNSCC cohort, respectively. The detailed information of the significantly changed metabolic enzymes was summarized in **Tables S3** and **S4**. The common DEMEs (23 upregulated and 9 downregulated) between GSE37991 and TCGA HNSCC cohort were shown in **Figure 1B**.

### Gene Ontology

**Figure 2:**
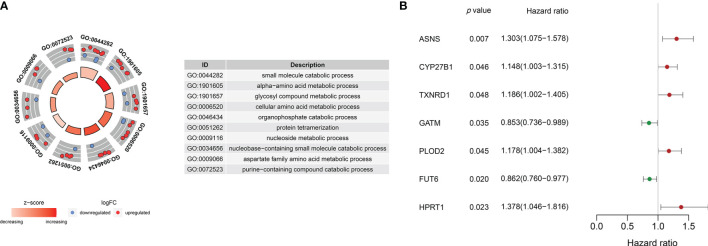
**(A)** Gene ontology analyses of the common DEMEs. **(B)** *ASNS*, *CYP27B1 TXNRD1*, *GATM*, *PLOD2*, *FUT6*, and *HPRT1* were significantly correlative with survival in the TCGA HNSCC cohort.

Gene ontology (GO) analysis of the DEMEs showed that small molecule catabolic process, alpha-amino acid metabolic process, glycosyl compound metabolic process, cellular amino acid metabolic process, organophosphate catabolic process, protein tetramerization, nucleoside metabolic process, nucleobase-containing small molecule catabolic process, aspartate family amino acid metabolic process, purine-containing compound catabolic process were the enriched (**Figure 2A**).

### Identification of the Prognostic Signature

**Figure 3:**
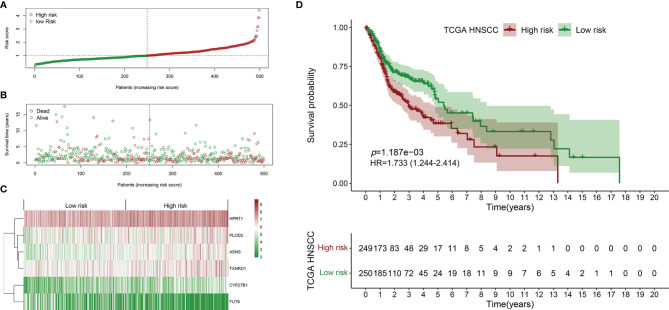
Establishment of the prognostic signature using the TCGA HNSCC cohort. **(A)** Scatter plot of the risk scores distribution. **(B)** Scatter plot of the OS and OS status distribution in the low‐and high‐risk groups. **(C)** The six prognostic DEMEs expression pattern between low‐ and high‐risk groups was shown in the heatmap. **(D)** The low-risk group got remarkably better OS than the high-risk group for the HNSCC patients.

**Figure 4:**
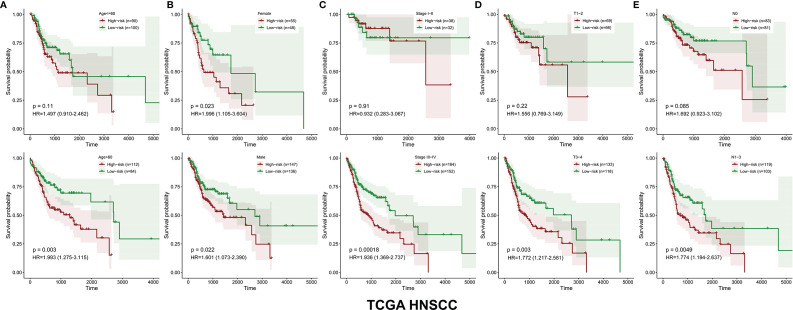
The OS distinction between the low‐ and high‐risk groups was classified by age, gender, clinical stage, T stage, and N stage in the TCGA HNSCC cohort **(A–E)**.

The survival-related DEMEs in the TCGA HNSCC cohort were identified by the univariate Cox regression, and *ASNS*, *CYP27B1*, *TXNRD1*, *GATM*, *PLOD2*, *FUT6*, and *HPRT1* were harvested. Based on the HRs, *GATM* and *FUT6* were protective genes, while *ASNS*, *CYP27B1*, *TXNRD1*, *PLOD2*, and *HPRT1* were risky genes (**Figure 2B**). The LASSO regression analysis identified six optimal DEMEs including *ASNS*, *CYP27B1*, *TXNRD1*, *PLOD2*, *FUT6*, and *HPRT1*. The risk score for each patient was computed as follows: risk score = (0.308) *ASNS + (0.228) * *CYP27B1* + (0.284) * *TXNRD1* + (0.174) * *PLOD2* + (-0.092) * *FUT6* + (0.362) * *HPRT1*. Subsequently, the HNSCC patients were divided into high- and low-risk groups based on the median value of risk scores (**Figure 3A**). The survival time and survival status of each HNSCC patient in TCGA HNSCC were presented in a scatter plot (**Figure 3B**). The expression levels of *ASNS*, *CYP27B1*, *TXNRD1*, *PLOD2*, *FUT6*, and *HPRT1* in each HNSCC patient were shown with a heatmap (**Figure 3C**). Besides, survival analysis demonstrated that the OS of the high-risk group was significantly lower compared to the low-risk group (**Figure 3D**). We then stratified the HNSCC patients with different clinical indices including age, gender, clinical stage, T stage, and N stage. As shown in **Figures 4A–E**, the low-risk group got remarkably better OS than the high-risk group for the HNSCC patients with age>60 (*p*=0.003), or with female gender (*p*=0.023) or with male gender (*p*=0.022), or at the clinical stage III-IV (*p*<0.001), or at the stage T3-4 (*p*=0.003), or with node metastasis (*p*=0.005).

### Validation of the Prognostic Signature in an Independent Cohort GSE65858

**Figure 5:**
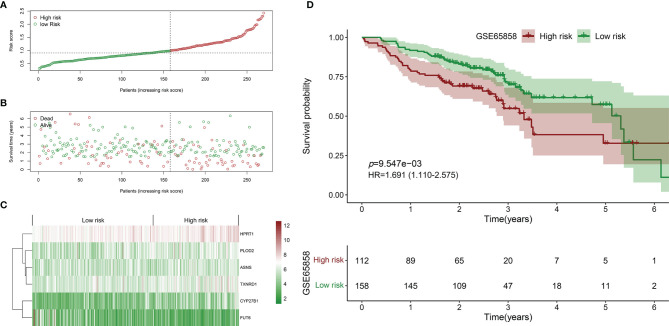
Validation of the prognostic biomarker using the GSE65858 validation cohort. **(A)** Scatter plot of the risk scores distribution. **(B)** Scatter plot of the OS and OS status distribution in the low‐and high‐risk groups. **(C)** The six prognostic DEMEs expression pattern between low‐ and high‐risk groups was shown in the heatmap. **(D)** The low-risk group got remarkably better OS than the high-risk group for the HNSCC patients.

**Figure 6:**
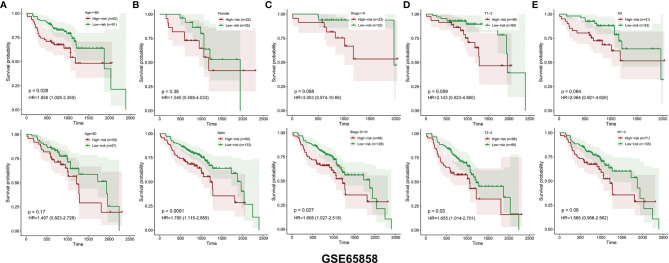
The OS distinction between the low‐ and high‐risk groups was classified by age, gender, clinical stage, T stage, and N stage in the GSE65858 validation cohort **(A–E)**.

Similarly, the HNSCC patients in GSE65858 were divided into high- and low-risk groups using the same median risk score in the TCGA HNSCC cohort (**Figure 5A**). The survival status, survival time, and expression level of prognosis-related DEMEs in each HNSCC patient were shown in **Figures 5B, C**. More importantly, the HNSCC patients in the high-risk group suffered a significantly poorer OS than those in the low-risk group (**Figure 5D**). As displayed in **Figures 6A–E**, the low-risk group got remarkably better OS than the high-risk group for the HNSCC patients with age ≤ 60 (*p*=0.026), or with male gender (*p*=0.009), or at the clinical stage III-IV (*p*=0.027), or the stage T3-4 (*p*=0.030).

### The Risk Score Is an Independent Prognostic Factor for HNSCC

**Figure 7:**
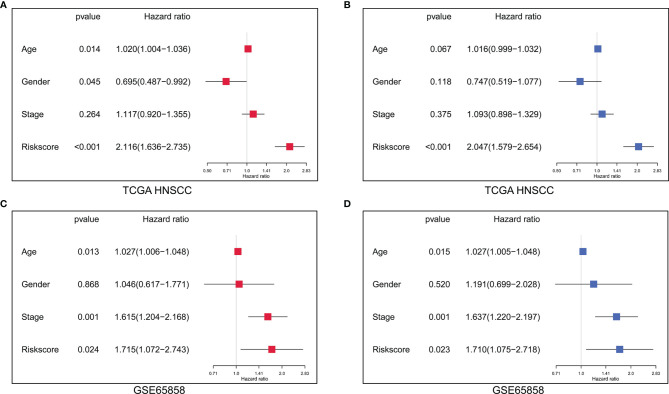
The independent prognostic factors were revealed by univariate and multivariate Cox regression analysis. **(A)** Age, gender and risk score were significantly correlated with survival in the TCGA HNSCC cohort by univariate Cox regression analysis. **(B)** The risk score was the independent prognostic indicator in the TCGA HNSCC cohort by multivariate Cox regression analysis. **(C)** Age, clinical stage and risk score were significantly associated with survival in the GSE65858 cohort by univariate Cox regression analysis. **(D)** Age, clinical stage and risk score were the independent prognostic indices in the GSE65858 cohort by multivariate Cox regression analysis.

In the TCGA HNSCC cohort, the univariate Cox regression analysis showed that age (*p* =0.014, HR=1.020), gender (*p* =0.045, HR=0.695) and risk score (*p*<0.001, HR=2.116) were significantly associated with survival (**Figure 7A**). The multivariate Cox regression analysis revealed that only risk score (*p*<0.001, HR=2.047) was the independent prognostic factor for HNSCC (**Figure 7B**). Similarly, in GSE65858, the univariate Cox regression analysis showed that age (*p* =0.013, HR=1.027), clinical stage (*p*=0.001, HR=1.615) and risk score (*p*=0.024, HR=1.715) were significantly correlated with survival (**Figure 7C**). The multivariate Cox regression analysis showed that age (*p* =0.015, HR=1.027), clinical stage (*p*=0.001, HR=1.637) and risk score (*p*=0.023, HR=1.710) were independent prognostic factors for HNSCC (**Figure 7D**).

### Nomogram Model Prediction

**Figure 8:**
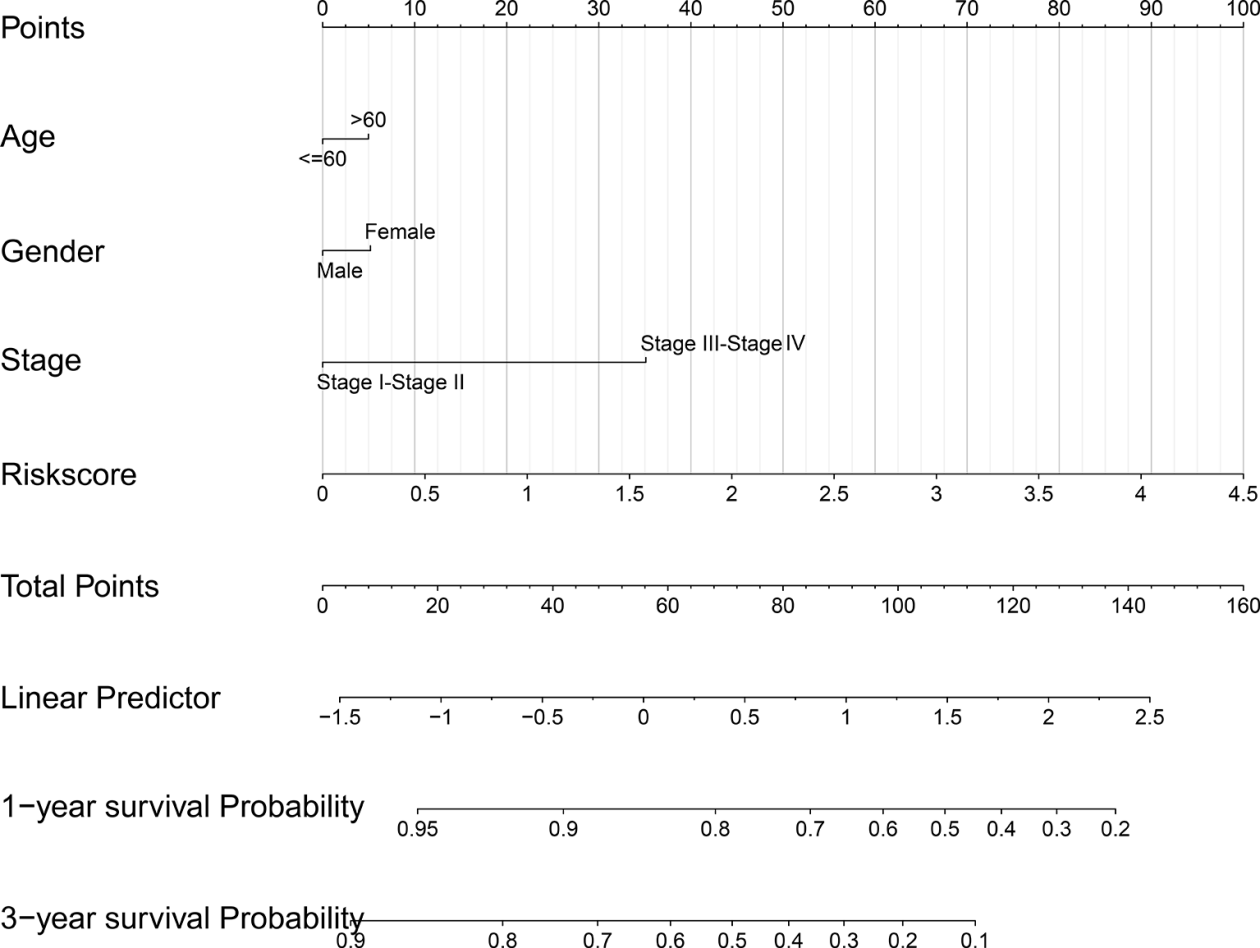
The nomogram prediction model was constructed with the risk score and other clinicopathological indices.

**Figure 9:**
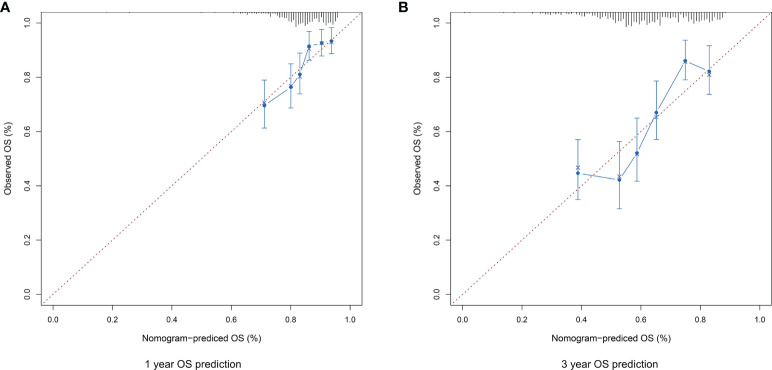
**(A)** The calibration plot for internal validation of the nomogram for 1-year OS prediction. **(B)** The calibration plot for internal validation of the nomogram for 3-year OS prediction.

The risk score, age, gender and clinical stage were incorporated into the nomogram model to forecast the clinical outcome of HNSCC (**Figure 8**). A total nomogram-based score was obtained for each HNSCC patient derived from the clinicopathological parameters and their corresponding points. The 1-year OS or 3-year OS of HNSCC patients was forecasted with the nomogram model. The calibration curves showed that the nomogram model we built up exhibited excellent conformance for predicting the 1-year OS or 3-year OS of HNSCC (**Figures 9A, B**). The C indices of the nomogram model are 0.658 or 0.616 for predicting the 1-year OS or 3-year OS of HNSCC, respectively.

### Knockdown of TXNRD1 Suppressed Malignant Activities of HNSCC Cells *In Vitro*

**Figure 10:**
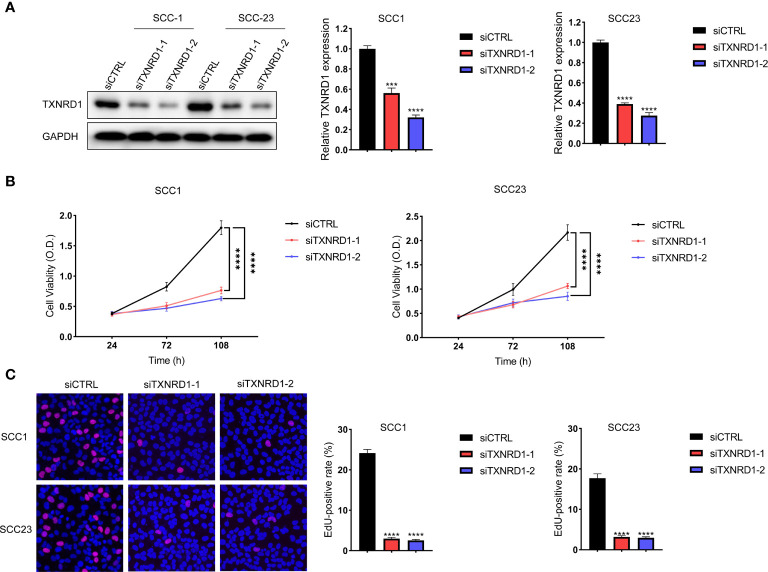
Knockdown of TXNRD1 suppressed malignant activities of HNSCC cells in vitro. **(A)** The expression level of TXNRD1 was significantly reduced in HNSCC cells following siTXNRD1 treatment. **(B)** Compared to the siCTRL treated cells, the OD values were markedly lower in TXNRD1-knockdown cells at indicated time points. **(C)** The EdU assay showed the percentage of EdU‐positive cells was dramatically lower in HNSCC cells treated with siTXNRD1. ***P < 0.001. ****P < 0.0001.

The expression level of TXNRD1 was significantly reduced in HNSCC cells following siTXNRD1 treatment (**Figure 10A**). Compared to the siCTRL treated cells, the MTT assay showed that the optical density (OD) values were markedly lower in TXNRD1-knockdown cells at indicated time points (**Figure 10B**). Similarly, the EdU assay showed the percentage of EdU‐positive cells was dramatically lower in HNSCC cells treated with siTXNRD1 (**Figure 10C**).

## Discussion

Metabolic reprogramming has been demonstrated to be essential for regulating the carcinogenesis of HNSCC. The energy consumption of neoplastic cells is increased to maintain their continuous growth and rapid proliferation (15). Nonetheless, the underlying molecular mechanisms for metabolic reprogramming in HNSCC are still unclear. Therefore, it’s urgently needed to figure out the potential metabolic enzymes that are correlative with the carcinogenesis of HNSCC. In this study, the common DEMEs between the TCGA HNSCC cohort and GSE37991 were identified. GO analyses of the common DEMEs revealed that the small molecule catabolic process, alpha-amino acid metabolic process, glycosyl compound metabolic process, etc., were enriched. In addition, a robust DEME-based prognostic signature including *HPRT1, PLOD2, ASNS, TXNRD1, CYP27B1*, and *FUT6* was constructed based on the TCGA HNSCC cohort. More importantly, this six-gene prognosis signature was successfully validated in another independent cohort GSE65858. Moreover, we have built up a robust risk score-based nomogram model which might provide personalized therapeutic guidance for treating HNSCC. *In vitro* experiment revealed that the knockdown of TXNRD1 suppressed malignant activities of HNSCC cells.

Based on our study, *ASNS*, *CYP27B1*, *TXNRD1*, *PLOD2*, *HPRT1* are identified as risky genes, and *FUT6* is deemed as the protective gene. ASNS catalyzed the ATP-dependent conversion of aspartate to asparagine, which promoted the proliferation of tumor cells through acting as an amino acid exchange factor (16). Downregulation of ASNS led to the suppression of asparagine synthesis by p53 and the unbalance between asparagine and aspartate, which subsequently inhibited the proliferation of neoplasm cells (17). Overexpression of ASNS facilitated the growth, metastasis, and chemoresistance of neoplasm cells, and a metabolic vulnerability was shown in specified cancer models with low-ASNS expression (18). CYP27B1, the vitamin D metabolizing enzyme, was upregulated at the beginning of the cancer carcinogenesis process with an increased expression of the vitamin D receptor (19). Besides, CYP27B1 may weaken the anticancer functions by locally altering the catabolic and anabolic progress of active vitamin D in cancer (20). On the contrary, CYP27B1 inhibited the proliferation, invasion, and migration of ovarian cancer cells *in vitro* (21). TXNRD1 is increased in head and neck cancer, breast cancer, and lung cancer, and its overexpression is correlative with poor prognosis (22, 23). Suppression of TXNRD1 inhibited the proliferation and induced apoptosis of hepatocellular carcinoma cells by modulating redox balance *in vitro* (24). In addition, TXNRD1 may promote DNA replication, tumorigenicity, and drug resistance by inducing the generation of reactive oxygen species (25). Increased expression of PLOD2 has been found in many types of cancer including breast cancer, colorectal cancer, hepatocellular carcinoma, esophageal squamous cell carcinoma, etc. (26) In HNSCC, PLOD2 is essential for the invasion and metastasis by activating the function of integrin β1 (27). In terms of mechanism, PLOD2 induced collagen cross-linking and maturation, and thus affected the biogenesis of the extracellular matrix of cancer-associated fibroblasts and stellate cells in the tumor microenvironment (26, 28). HPRT1 is located on chromosome X and supplies recycled nucleotides to the cell cycle for DNA and RNA synthesis (29). Increased expression of HPRT1 was observed in many cancer types, indicating that HPRT1 may be a potential diagnostic and prognostic marker (30). FUT6 produced glycans for tumor cells *via* the PI3K/Akt signaling pathway, which was regulated by miR-125a-3p in colorectal cancer (31). Overexpression of FUT6 inhibited the malignant activities of neoplasm cells by suppressing the dimerization and phosphorylation of epidermal growth factor receptors (32).

Our robust metabolic enzyme-based risk signature has several advantages compared to the gene model with existing signatures. Firstly, to the best of our knowledge, currently few metabolic enzyme-based prognostic signatures are available for predicting the prognosis of HNSCC. Secondly, most prognostic signatures included up to 10 genes, which might be not facilitated for clinical application. Our model has simplified the number of genes in the risk signature to six. Thirdly, compared to many existing prognostic signatures, we have successfully constructed a metabolic enzyme-based nomogram model which showed great promise for therapeutic guidance for HNSCC. Fourthly, the multivariate analysis showed that metabolic enzyme-based prognostic signature was more robust for predicting the prognosis of HNSCC compared to the TNM stage.

Although our study might provide clinical guidance for treating HNSCC, several limitations are needed to be considered. Firstly, the detailed clinicopathological information such as M stage and HPV infection status is missing in most HNSCC patients. Therefore, the importance of these clinicopathological parameters couldn’t be included in the nomogram model. Secondly, most patients in the TCGA HNSCC cohort are whites. The effectiveness of the prognostic signature in other races is warranted for further validation. Thirdly, the AUC value of our metabolic-enzyme based risk signature was not high (data not shown), and needs further improvement. However, it is very difficult to use a risk model or a panel of biomarkers to accurately predict the prognosis of HNSCC. In the clinical setting, many clinicopathological parameters such as clinical symptoms, psychological condition and systemic diseases should be combined to comprehensively evaluate the clinical outcome of HNSCC. Finally, large-scale cohorts are needed to verify our DEME-based prognostic signature.

## Conclusions

In summary, our study has identified the common DEMEs between HNSCC and normal controls, which may be correlative with the initiation and development of HNSCC. In addition, we have successfully built up and validated a robust DEME-based prognostic signature. Moreover, the nomogram model might provide useful guidance for the precision treatment of HNSCC.

## Data Availability Statement

The datasets presented in this study can be found in online repositories. The names of the repository/repositories and accession number(s) can be found in the article/**Supplementary Material**.

## Author Contributions

Conceptualization and funding acquisition, XZ and LC. ZM, XZ, and LC designed and coordinated the study. ZM and HC performed the statistical analysis. HC and MH participated in the collection of database and analysis tools. ZM, XZ, and LC drafted the paper. All authors have read and agreed to the submitted version of the manuscript.

## Funding

This work was supported by the National Natural Science Foundation of China (81901006). Guangdong Basic and Applied Basic Research Foundation (2020A1515110051).

## Conflict of Interest

The authors declare that the research was conducted in the absence of any commercial or financial relationships that could be construed as a potential conflict of interest.

## Publisher’s Note

All claims expressed in this article are solely those of the authors and do not necessarily represent those of their affiliated organizations, or those of the publisher, the editors and the reviewers. Any product that may be evaluated in this article, or claim that may be made by its manufacturer, is not guaranteed or endorsed by the publisher.

## Abbreviations

HNSCC, Head and neck squamous cell carcinoma; TCGA, The Cancer Genome Atlas; DEME, differentially expressed metabolic enzyme; HPV, human papillomavirus; OS, overall survival; GEO, Gene Expression Omnibus; DEG, differentially expressed gene; GO, Gene Ontology; LASSO, least absolute shrinkage and selection operator; RSN, Robust Spline Normalization; PVDF, polyvinylidene difluoride; MTT, 3-(4, 5-dimethylthiazol-2-yl)-2, 5-diphenyltetrazolium bromide; DMSO, Dimethyl sulfoxide; EdU, 5‐Ethynyl‐2′‐deoxyuridine; AUC, area under the curve; OD, optical density.
