# Supplementary material for: A Robust Metabolic Enzyme-Based Prognostic Signature for Head and Neck Squamous Cell Carcinoma
Source: Front Oncol. 2022 Jan 20;11:770241. doi: 10.3389/fonc.2021.770241 (PMC8810637; doi:10.3389/fonc.2021.770241)
Supplement: Supplementary file 1 [file Table_1.docx]

**Supplementary Table 1** The clinical information of the TCGA HNSCC cohort

| Clinicopathological features | Number |
| --- | --- |
| Age |  |
| Mean (SD) | 60.84 (11.63) |
| Gender, n (%) |  |
| Male | 283 (73.32%) |
| Female | 103 (26.68%) |
| Pathological diagnosis |  |
| Squamous cell carcinoma | 386 (100%) |
| TNM stage |  |
| Stage I | 18 (4.66%) |
| Stage II | 62 (16.06%) |
| Stage III | 91 (23.58%) |
| Stage IV | 215 (55.70%) |
