# Supplementary material for: A Robust Metabolic Enzyme-Based Prognostic Signature for Head and Neck Squamous Cell Carcinoma
Source: Front Oncol. 2022 Jan 20;11:770241. doi: 10.3389/fonc.2021.770241 (PMC8810637; doi:10.3389/fonc.2021.770241)
Supplement: Supplementary file 2 [file Table_2.docx]

**Supplementary Table 2** The clinical information of the GSE65858 validation cohort

| Clinicopathological features | Number |
| --- | --- |
| Age |  |
| Mean (SD) | 60.12 (10.33) |
| Gender, n (%) |  |
| Male | 223 (82.59%) |
| Female | 47 (17.41%) |
| Pathological diagnosis |  |
| Squamous cell carcinoma | 270 (100%) |
| TNM stage |  |
| Stage I | 18 (6.67%) |
| Stage II | 37 (13.70%) |
| Stage III | 37 (13.70%) |
| Stage IV | 178 (65.93%) |
