# Supplementary material for: A Robust Metabolic Enzyme-Based Prognostic Signature for Head and Neck Squamous Cell Carcinoma
Source: Front Oncol. 2022 Jan 20;11:770241. doi: 10.3389/fonc.2021.770241 (PMC8810637; doi:10.3389/fonc.2021.770241)
Supplement: Supplementary file 3 [file Table_3.docx]

**Supplementary Table 3** Significantly changed metabolic enzymes in TCGA HNSCC cohort.

| Genes names | logFC | PValue | FDR |
| --- | --- | --- | --- |
| GPD1 | -4.81752 | 2.57E-131 | 3.17E-128 |
| AWAT2 | -6.11108 | 1.40E-106 | 8.63E-104 |
| GPD1L | -2.48835 | 3.08E-104 | 1.26E-101 |
| FBP2 | -5.10025 | 4.47E-102 | 1.38E-99 |
| ADH1B | -4.90256 | 8.29E-101 | 2.05E-98 |
| ATP6V0A4 | -3.78337 | 2.72E-86 | 5.59E-84 |
| ENO3 | -4.69273 | 2.29E-82 | 4.04E-80 |
| COX7A1 | -3.38428 | 4.32E-81 | 6.67E-79 |
| ATP6V1B1 | -4.07556 | 6.92E-80 | 9.49E-78 |
| GPT | -2.93834 | 4.44E-71 | 5.47E-69 |
| PYGM | -5.63696 | 2.65E-70 | 2.97E-68 |
| COQ8A | -2.60888 | 1.28E-66 | 1.31E-64 |
| PDK4 | -3.93468 | 3.93E-64 | 3.73E-62 |
| OBSCN | -2.95749 | 5.56E-64 | 4.90E-62 |
| HMGCS2 | -4.26046 | 2.12E-61 | 1.74E-59 |
| COLGALT1 | 1.619201 | 3.12E-57 | 2.40E-55 |
| ETFDH | -1.34231 | 5.63E-56 | 4.08E-54 |
| PHYH | -1.98807 | 1.43E-53 | 9.83E-52 |
| GMDS | -1.46085 | 2.64E-53 | 1.71E-51 |
| PFKM | -1.79992 | 1.35E-51 | 8.33E-50 |
| PHKG1 | -3.25354 | 2.73E-51 | 1.60E-49 |
| HSD11B2 | -2.11719 | 2.94E-50 | 1.65E-48 |
| AMPD1 | -4.02205 | 2.33E-47 | 1.25E-45 |
| ACOX2 | -2.25619 | 4.85E-47 | 2.49E-45 |
| GOT1 | -1.15336 | 5.96E-45 | 2.94E-43 |
| CKMT2 | -3.88956 | 1.55E-44 | 7.09E-43 |
| ACADM | -1.28543 | 3.76E-44 | 1.66E-42 |
| LDHD | -2.12708 | 1.49E-42 | 6.34E-41 |
| LPIN1 | -1.49258 | 5.08E-42 | 2.09E-40 |
| TK1 | 1.945803 | 4.38E-41 | 1.74E-39 |
| GPX3 | -2.32003 | 3.49E-39 | 1.34E-37 |
| ACACB | -1.97159 | 7.45E-39 | 2.78E-37 |
| ALDH9A1 | -1.12946 | 6.35E-38 | 2.30E-36 |
| ATP1A2 | -3.98932 | 1.54E-37 | 5.43E-36 |
| MGLL | -1.80927 | 1.69E-37 | 5.79E-36 |
| ACAT1 | -1.23336 | 1.97E-37 | 6.57E-36 |
| ADHFE1 | -2.18509 | 4.74E-37 | 1.54E-35 |
| CARNS1 | -2.50476 | 1.14E-36 | 3.60E-35 |
| GPT2 | -1.56328 | 2.71E-36 | 8.34E-35 |
| ACADSB | -1.48413 | 4.65E-36 | 1.40E-34 |
| CKM | -4.42805 | 1.52E-35 | 4.47E-34 |
| ENPP4 | -2.17064 | 1.25E-32 | 3.41E-31 |
| CRAT | -1.93488 | 1.27E-32 | 3.41E-31 |
| AOX1 | -2.16144 | 4.77E-32 | 1.22E-30 |
| PLOD3 | 1.862974 | 2.53E-31 | 6.36E-30 |
| PRIM2 | 1.355012 | 2.93E-31 | 7.23E-30 |
| FUT6 | -2.97442 | 3.07E-31 | 7.43E-30 |
| ADSSL1 | -1.9291 | 4.32E-31 | 1.02E-29 |
| GSTA2 | -3.91865 | 4.41E-31 | 1.03E-29 |
| MT-ND1 | -1.35338 | 3.63E-30 | 8.13E-29 |
| PRKAA2 | -2.54729 | 3.86E-30 | 8.49E-29 |
| ATAD2 | 1.643895 | 7.50E-29 | 1.57E-27 |
| COX6A2 | -4.07284 | 1.14E-28 | 2.34E-27 |
| HPRT1 | 1.059078 | 1.45E-28 | 2.88E-27 |
| CHST11 | 2.145912 | 4.40E-28 | 8.34E-27 |
| PLOD1 | 1.618537 | 5.24E-28 | 9.78E-27 |
| ENO2 | 2.736593 | 1.10E-27 | 2.02E-26 |
| MT-ND5 | -1.4538 | 1.33E-27 | 2.41E-26 |
| LPCAT1 | 1.802439 | 1.57E-27 | 2.81E-26 |
| PGM1 | -1.08334 | 1.68E-27 | 2.97E-26 |
| GAMT | -1.74477 | 4.02E-27 | 6.97E-26 |
| PNPLA7 | -1.7343 | 1.52E-26 | 2.56E-25 |
| DNMT3B | 2.362412 | 2.34E-26 | 3.89E-25 |
| CHST9 | -3.17404 | 2.39E-26 | 3.93E-25 |
| MGME1 | 1.064333 | 3.19E-26 | 5.12E-25 |
| ALDH6A1 | -1.1819 | 7.02E-26 | 1.10E-24 |
| MT-ND6 | -1.41353 | 1.83E-25 | 2.82E-24 |
| ACPP | -1.84156 | 2.09E-25 | 3.19E-24 |
| DGAT2 | -1.84966 | 5.95E-25 | 8.95E-24 |
| P4HA1 | 1.570118 | 1.30E-24 | 1.94E-23 |
| GMPR | -1.80315 | 1.41E-24 | 2.06E-23 |
| CLYBL | -1.33791 | 1.09E-23 | 1.58E-22 |
| ACOT7 | 1.318027 | 1.13E-23 | 1.62E-22 |
| GNE | -1.43799 | 2.27E-23 | 3.22E-22 |
| ACYP2 | -1.03639 | 2.68E-23 | 3.75E-22 |
| HSD17B6 | 2.082985 | 3.54E-23 | 4.90E-22 |
| MT-CO1 | -1.08223 | 3.68E-23 | 5.04E-22 |
| POLD1 | 1.137191 | 6.38E-23 | 8.65E-22 |
| EPHX2 | -1.50139 | 6.92E-23 | 9.27E-22 |
| ADA | 1.691909 | 9.77E-23 | 1.30E-21 |
| ALDH1L1 | -2.45183 | 1.13E-22 | 1.46E-21 |
| PRG4 | -2.86683 | 1.14E-22 | 1.46E-21 |
| MTHFD1L | 1.27872 | 1.97E-22 | 2.50E-21 |
| SOD3 | -1.50574 | 7.85E-22 | 9.49E-21 |
| FUT3 | -1.8774 | 8.11E-22 | 9.71E-21 |
| ST6GALNAC1 | -2.0874 | 9.41E-22 | 1.12E-20 |
| AK7 | -1.93805 | 1.06E-21 | 1.24E-20 |
| POLA2 | 1.098104 | 1.78E-21 | 2.07E-20 |
| SMS | 1.142544 | 2.74E-21 | 3.15E-20 |
| FKBP9 | 1.280957 | 1.08E-20 | 1.21E-19 |
| FUT2 | -1.40982 | 2.08E-20 | 2.27E-19 |
| ALDH3B1 | -1.39308 | 2.24E-20 | 2.42E-19 |
| LPL | -1.98276 | 3.03E-20 | 3.25E-19 |
| NNT | -1.14028 | 4.19E-20 | 4.41E-19 |
| CHST2 | 2.6107 | 4.61E-20 | 4.82E-19 |
| IMPDH1 | 1.234859 | 2.86E-19 | 2.89E-18 |
| DNMT1 | 1.090894 | 3.03E-19 | 3.04E-18 |
| ACHE | -2.02002 | 4.72E-19 | 4.70E-18 |
| P4HA2 | 1.497086 | 5.33E-19 | 5.22E-18 |
| POLR2H | 1.050182 | 5.46E-19 | 5.30E-18 |
| CYP27B1 | 2.512013 | 7.83E-19 | 7.42E-18 |
| ATP1B3 | 1.144727 | 1.68E-18 | 1.55E-17 |
| CHPT1 | -1.49539 | 1.81E-18 | 1.66E-17 |
| MT-ND3 | -1.02902 | 1.83E-18 | 1.66E-17 |
| APOBEC2 | -3.08989 | 2.00E-18 | 1.80E-17 |
| RRM2 | 1.180984 | 7.60E-18 | 6.60E-17 |
| PLOD2 | 1.708976 | 8.14E-18 | 7.02E-17 |
| TYMS | 1.388388 | 9.51E-18 | 8.09E-17 |
| DTYMK | 1.06288 | 1.04E-17 | 8.74E-17 |
| GATM | -1.51361 | 1.88E-17 | 1.56E-16 |
| AK1 | -1.13425 | 1.89E-17 | 1.56E-16 |
| NUDT1 | 1.245122 | 2.84E-17 | 2.32E-16 |
| SUGCT | 2.034378 | 4.07E-17 | 3.26E-16 |
| PLA2G7 | 1.978995 | 4.76E-17 | 3.76E-16 |
| ACSL1 | -1.04577 | 5.37E-17 | 4.22E-16 |
| CHST7 | 1.884008 | 5.42E-17 | 4.23E-16 |
| CHST15 | 1.298151 | 5.56E-17 | 4.31E-16 |
| MACROD1 | -1.14219 | 6.38E-17 | 4.89E-16 |
| POLQ | 1.597761 | 7.10E-17 | 5.40E-16 |
| POLE2 | 1.1803 | 4.13E-16 | 3.03E-15 |
| ACER1 | -2.37169 | 7.09E-16 | 5.15E-15 |
| RSAD2 | 2.597109 | 7.96E-16 | 5.74E-15 |
| ATP8A1 | -1.38002 | 8.81E-16 | 6.31E-15 |
| GNMT | -1.50516 | 9.38E-16 | 6.68E-15 |
| ACP5 | 1.316318 | 1.72E-15 | 1.20E-14 |
| CES2 | -1.06128 | 3.69E-15 | 2.47E-14 |
| AOC3 | -1.38741 | 4.10E-15 | 2.72E-14 |
| PDE7A | 1.221568 | 7.39E-15 | 4.82E-14 |
| ATP6V1C2 | -1.69331 | 8.08E-15 | 5.22E-14 |
| NAGS | 1.495925 | 9.73E-15 | 6.22E-14 |
| PRIM1 | 1.008106 | 9.98E-15 | 6.34E-14 |
| TYMP | 1.565872 | 1.05E-14 | 6.62E-14 |
| GPX8 | 1.379977 | 1.08E-14 | 6.79E-14 |
| GLS | 1.078112 | 2.28E-14 | 1.39E-13 |
| FKBP14 | 1.139059 | 3.12E-14 | 1.89E-13 |
| FKBP10 | 1.963673 | 3.77E-14 | 2.25E-13 |
| PLD6 | 1.363612 | 5.74E-14 | 3.41E-13 |
| PTGDS | -1.65255 | 5.79E-14 | 3.41E-13 |
| AADAC | -2.32835 | 5.84E-14 | 3.43E-13 |
| ACAA2 | -1.06861 | 1.42E-13 | 8.21E-13 |
| NOS1 | -1.79807 | 2.13E-13 | 1.22E-12 |
| PLA2G2A | -2.42724 | 3.90E-13 | 2.19E-12 |
| NSUN7 | -1.38197 | 4.04E-13 | 2.25E-12 |
| CMPK2 | 1.887374 | 4.27E-13 | 2.36E-12 |
| ALOX15 | -2.5286 | 5.04E-13 | 2.75E-12 |
| ME3 | -1.18488 | 6.54E-13 | 3.52E-12 |
| NUDT11 | 2.132946 | 7.58E-13 | 4.06E-12 |
| IL4I1 | 1.737482 | 2.46E-12 | 1.26E-11 |
| ODC1 | 1.930951 | 3.38E-12 | 1.70E-11 |
| CYP11A1 | -1.91679 | 5.44E-12 | 2.68E-11 |
| ARSJ | 1.483334 | 5.70E-12 | 2.80E-11 |
| P4HA3 | 2.20446 | 8.17E-12 | 3.93E-11 |
| HNMT | -1.0072 | 1.21E-11 | 5.78E-11 |
| PLA2G16 | -1.36847 | 1.57E-11 | 7.41E-11 |
| HKDC1 | 3.304811 | 1.80E-11 | 8.45E-11 |
| ASNS | 1.145187 | 3.91E-11 | 1.81E-10 |
| RDH16 | 1.937385 | 4.83E-11 | 2.21E-10 |
| ENTPD7 | 1.092159 | 4.93E-11 | 2.25E-10 |
| PTGIS | -1.74356 | 5.49E-11 | 2.49E-10 |
| FOXRED2 | 1.238194 | 6.21E-11 | 2.80E-10 |
| DDAH1 | -1.0559 | 6.40E-11 | 2.87E-10 |
| ALDH1A3 | -1.2641 | 1.40E-10 | 6.10E-10 |
| HSD11B1 | -1.35802 | 1.44E-10 | 6.26E-10 |
| AKR1E2 | 1.594104 | 1.91E-10 | 8.21E-10 |
| RPE65 | 3.490097 | 2.56E-10 | 1.08E-09 |
| HK3 | 1.82234 | 2.73E-10 | 1.15E-09 |
| SULT2B1 | -1.38756 | 2.80E-10 | 1.18E-09 |
| SMPD3 | -1.37393 | 2.88E-10 | 1.20E-09 |
| NT5E | 1.785384 | 3.51E-10 | 1.45E-09 |
| ASPG | -1.39195 | 3.82E-10 | 1.57E-09 |
| BCAT1 | 1.754053 | 4.68E-10 | 1.89E-09 |
| CDO1 | -1.55872 | 4.82E-10 | 1.94E-09 |
| ATP1B4 | -2.07811 | 5.20E-10 | 2.08E-09 |
| ENPP5 | -1.52239 | 5.61E-10 | 2.23E-09 |
| ARSI | 1.735003 | 7.30E-10 | 2.86E-09 |
| PYGL | 1.165706 | 1.21E-09 | 4.60E-09 |
| CHDH | -1.44007 | 2.23E-09 | 8.34E-09 |
| CHST6 | -1.36198 | 2.99E-09 | 1.11E-08 |
| PRTFDC1 | 1.181055 | 3.12E-09 | 1.15E-08 |
| KYNU | 1.76756 | 3.53E-09 | 1.30E-08 |
| ALDH3A1 | -1.68962 | 3.87E-09 | 1.42E-08 |
| SCD5 | 1.016862 | 4.76E-09 | 1.73E-08 |
| ECHDC3 | -1.24016 | 1.10E-08 | 3.88E-08 |
| MAOB | -1.41722 | 2.69E-08 | 9.16E-08 |
| PDE4B | -1.07445 | 2.71E-08 | 9.20E-08 |
| SMPD5 | 1.812003 | 5.79E-08 | 1.92E-07 |
| ADH7 | -1.65313 | 8.16E-08 | 2.66E-07 |
| MIOX | 2.8611 | 9.94E-08 | 3.21E-07 |
| AKR1C3 | 2.080539 | 1.07E-07 | 3.46E-07 |
| GPX7 | 1.124437 | 1.11E-07 | 3.57E-07 |
| COX6B2 | 1.673186 | 1.53E-07 | 4.79E-07 |
| LRAT | 2.396349 | 2.59E-07 | 7.85E-07 |
| DHCR7 | 1.13104 | 2.75E-07 | 8.31E-07 |
| PTGS2 | 1.764536 | 2.81E-07 | 8.48E-07 |
| AKR1C1 | 2.101819 | 7.28E-07 | 2.07E-06 |
| TXNRD1 | 1.093287 | 7.56E-07 | 2.15E-06 |
| AMDHD1 | 2.238568 | 2.54E-06 | 6.95E-06 |
| TDO2 | 1.427599 | 2.71E-06 | 7.39E-06 |
| ALDH1A1 | -1.39292 | 2.87E-06 | 7.73E-06 |
| ARSE | 1.430132 | 4.17E-06 | 1.11E-05 |
| CD38 | 1.134422 | 4.79E-06 | 1.26E-05 |
| CHST1 | 1.225533 | 5.31E-06 | 1.39E-05 |
| TH | 2.370228 | 8.38E-06 | 2.12E-05 |
| COX7B2 | 3.880341 | 1.02E-05 | 2.54E-05 |
| AKR1C2 | 1.678936 | 1.34E-05 | 3.32E-05 |
| EPHX3 | -1.22535 | 2.35E-05 | 5.64E-05 |
| DGKG | 1.32514 | 4.17E-05 | 9.64E-05 |
| B3GALT5 | -1.32704 | 5.44E-05 | 0.000123513 |
| SULT1E1 | 1.794309 | 7.99E-05 | 0.000177115 |
| UGT1A8 | -1.70331 | 8.94E-05 | 0.000196826 |
| TKTL1 | 4.03366 | 0.000131394 | 0.000282739 |
| LIPG | 1.157885 | 0.000224405 | 0.000468969 |
| PLA2G2D | 1.571123 | 0.000354889 | 0.000724468 |
| HSD17B3 | 1.326357 | 0.000402803 | 0.00081419 |
| DHRS2 | 1.783735 | 0.000568809 | 0.001131195 |
| ATP6V0D2 | 1.042487 | 0.000600872 | 0.001189207 |
| PLA2G2F | 1.257798 | 0.001289536 | 0.002423777 |
| CEL | 1.457752 | 0.002056242 | 0.003739449 |
| ACP7 | 1.130468 | 0.002175902 | 0.00393963 |
| PCYT1B | 1.09026 | 0.003828087 | 0.00670459 |
| ARG1 | -1.1601 | 0.004084847 | 0.007103831 |
| GSTA1 | -1.25265 | 0.00606585 | 0.010189636 |
| NUDT10 | 1.065333 | 0.00702261 | 0.011664541 |
