# Supplementary material for: A Robust Metabolic Enzyme-Based Prognostic Signature for Head and Neck Squamous Cell Carcinoma
Source: Front Oncol. 2022 Jan 20;11:770241. doi: 10.3389/fonc.2021.770241 (PMC8810637; doi:10.3389/fonc.2021.770241)
Supplement: Supplementary file 4 [file Table_4.docx]

**Supplementary Table 4** Significantly changed metabolic enzymes in GSE37991 cohort.

| Genes names | logFC | PValue | FDR |
| --- | --- | --- | --- |
| GATM | -2.70955 | 8.60E-47 | 1.12E-43 |
| CROT | 3.451377 | 2.07E-42 | 1.35E-39 |
| PGAM2 | -3.00142 | 1.07E-38 | 4.62E-36 |
| ATP5J | 4.896824 | 2.42E-37 | 7.85E-35 |
| B3GALT6 | 2.594526 | 7.39E-35 | 1.92E-32 |
| HMGCLL1 | -2.18796 | 5.20E-34 | 1.13E-31 |
| B4GALT5 | 3.267033 | 1.27E-33 | 2.36E-31 |
| ARSE | -2.37739 | 2.78E-32 | 4.51E-30 |
| GNPDA1 | 5.741062 | 5.12E-30 | 7.40E-28 |
| GCSH | -1.73337 | 4.60E-29 | 5.98E-27 |
| ATP8A1 | 1.926806 | 1.12E-28 | 1.33E-26 |
| SRM | 4.462144 | 2.17E-28 | 2.36E-26 |
| PDP2 | 2.234398 | 8.10E-28 | 7.77E-26 |
| B3GALT5 | -2.38011 | 8.36E-28 | 7.77E-26 |
| LHPP | 4.249934 | 5.87E-27 | 5.08E-25 |
| COQ5 | 5.761708 | 7.61E-27 | 6.18E-25 |
| COX6B1 | 5.353962 | 1.43E-26 | 1.09E-24 |
| GAPDH | 4.271425 | 7.28E-26 | 5.26E-24 |
| NDUFA8 | 2.612555 | 9.55E-26 | 6.53E-24 |
| ATP6V1E1 | 3.971795 | 1.05E-25 | 6.85E-24 |
| COX6C | 4.807049 | 1.42E-25 | 8.36E-24 |
| ALDOC | 5.368538 | 1.42E-25 | 8.36E-24 |
| ATP6V0E1 | 5.239661 | 1.68E-25 | 9.49E-24 |
| ALDH1A3 | 4.088118 | 2.39E-25 | 1.25E-23 |
| ALDH2 | 5.217675 | 2.41E-25 | 1.25E-23 |
| NDUFA12 | 5.291026 | 2.75E-25 | 1.37E-23 |
| TFB1M | 5.093194 | 3.60E-25 | 1.73E-23 |
| IDH3A | 5.104167 | 6.11E-25 | 2.76E-23 |
| ATP6V1A | 5.263403 | 6.15E-25 | 2.76E-23 |
| NDUFB7 | 5.097759 | 6.86E-25 | 2.97E-23 |
| MTHFS | 3.975752 | 7.98E-25 | 3.29E-23 |
| ATP5O | 5.045847 | 8.20E-25 | 3.29E-23 |
| GSTO1 | 5.283103 | 8.34E-25 | 3.29E-23 |
| PLA2G4B | 5.242737 | 9.12E-25 | 3.48E-23 |
| LPIN1 | 5.067134 | 9.61E-25 | 3.48E-23 |
| ATP1B3 | 5.048669 | 9.64E-25 | 3.48E-23 |
| GALK1 | 5.085217 | 1.06E-24 | 3.72E-23 |
| MBOAT2 | 1.789579 | 1.14E-24 | 3.89E-23 |
| ACAD9 | 4.032775 | 1.34E-24 | 4.47E-23 |
| COX17 | 5.192127 | 1.49E-24 | 4.85E-23 |
| ABHD10 | 4.924661 | 1.94E-24 | 6.15E-23 |
| PAPD4 | 3.932778 | 2.05E-24 | 6.33E-23 |
| NDUFB2 | 3.882845 | 2.10E-24 | 6.33E-23 |
| DGUOK | 4.946081 | 2.14E-24 | 6.33E-23 |
| ABHD6 | 5.04465 | 2.22E-24 | 6.42E-23 |
| ETFDH | 4.383829 | 2.28E-24 | 6.43E-23 |
| CYB5R1 | 1.434253 | 2.60E-24 | 7.18E-23 |
| CYB5R3 | 4.17306 | 3.18E-24 | 8.61E-23 |
| SQLE | 4.81821 | 4.30E-24 | 1.14E-22 |
| MGST3 | 4.515295 | 5.67E-24 | 1.45E-22 |
| ATP2B4 | 4.211702 | 5.70E-24 | 1.45E-22 |
| SEPHS1 | 4.773841 | 7.05E-24 | 1.76E-22 |
| PLOD3 | 4.603502 | 1.32E-23 | 3.24E-22 |
| STEAP4 | 3.150904 | 1.36E-23 | 3.28E-22 |
| TK1 | 4.45578 | 2.05E-23 | 4.84E-22 |
| RSAD2 | 4.61729 | 2.49E-23 | 5.78E-22 |
| ENDOG | -2.28773 | 2.59E-23 | 5.90E-22 |
| VPS29 | 4.708119 | 2.64E-23 | 5.93E-22 |
| PPCS | 4.840619 | 2.94E-23 | 6.48E-22 |
| CYB5R4 | 4.672492 | 3.05E-23 | 6.54E-22 |
| HPRT1 | 4.886818 | 3.07E-23 | 6.54E-22 |
| GSS | 2.011644 | 3.21E-23 | 6.74E-22 |
| GUSB | 4.330828 | 4.19E-23 | 8.64E-22 |
| HSD17B8 | 1.901183 | 6.67E-23 | 1.34E-21 |
| G6PC3 | 4.490939 | 6.68E-23 | 1.34E-21 |
| ASNS | 4.314381 | 8.49E-23 | 1.67E-21 |
| TFB2M | 4.4539 | 1.06E-22 | 2.05E-21 |
| TGDS | 2.310112 | 1.31E-22 | 2.51E-21 |
| GBA2 | -2.62888 | 1.42E-22 | 2.68E-21 |
| ATP6V1D | 4.596763 | 1.73E-22 | 3.21E-21 |
| PLA2G7 | 3.289028 | 1.84E-22 | 3.37E-21 |
| TXNDC5 | 2.427872 | 2.27E-22 | 4.09E-21 |
| ALDH3A2 | 2.943072 | 2.43E-22 | 4.33E-21 |
| GSTA4 | 4.255399 | 2.49E-22 | 4.37E-21 |
| ATP5C1 | 4.757449 | 2.66E-22 | 4.61E-21 |
| ELAC2 | -1.68556 | 2.74E-22 | 4.66E-21 |
| HMGCS1 | 4.244494 | 2.76E-22 | 4.66E-21 |
| SDHB | 4.839188 | 3.19E-22 | 5.31E-21 |
| FOXRED1 | 4.129568 | 3.33E-22 | 5.44E-21 |
| ATP5I | 4.871876 | 3.35E-22 | 5.44E-21 |
| GSTM3 | 4.131004 | 3.48E-22 | 5.58E-21 |
| BPGM | 2.389206 | 3.52E-22 | 5.58E-21 |
| ATP6V1C1 | 4.132726 | 3.71E-22 | 5.81E-21 |
| GART | 3.902987 | 4.88E-22 | 7.56E-21 |
| CES2 | 4.164699 | 5.61E-22 | 8.58E-21 |
| AUH | 3.744868 | 5.89E-22 | 8.91E-21 |
| ME1 | 3.905393 | 6.93E-22 | 1.04E-20 |
| RBKS | 1.780341 | 7.67E-22 | 1.13E-20 |
| METTL4 | 2.856702 | 8.51E-22 | 1.24E-20 |
| CYP27B1 | 2.888119 | 1.36E-21 | 1.96E-20 |
| ATP6V0A2 | 4.029738 | 1.68E-21 | 2.41E-20 |
| HSD17B4 | 3.418111 | 1.76E-21 | 2.48E-20 |
| GMPR2 | 3.892378 | 2.60E-21 | 3.63E-20 |
| POLR2G | 3.841207 | 2.82E-21 | 3.90E-20 |
| GRHPR | 3.659872 | 3.22E-21 | 4.35E-20 |
| GBE1 | 2.33002 | 3.23E-21 | 4.35E-20 |
| RPA1 | 3.362535 | 3.25E-21 | 4.35E-20 |
| DECR1 | 3.801701 | 3.41E-21 | 4.52E-20 |
| SDHA | 3.954863 | 3.47E-21 | 4.56E-20 |
| NT5DC1 | 3.72839 | 3.89E-21 | 5.06E-20 |
| NDUFS8 | 3.104312 | 4.32E-21 | 5.51E-20 |
| PNPLA8 | 3.83644 | 4.32E-21 | 5.51E-20 |
| PDSS2 | 3.355118 | 5.11E-21 | 6.45E-20 |
| PDK2 | -2.90147 | 5.26E-21 | 6.58E-20 |
| NDUFB11 | 3.66623 | 6.14E-21 | 7.60E-20 |
| TRIT1 | 3.709873 | 1.01E-20 | 1.24E-19 |
| ADCY3 | 3.981773 | 1.05E-20 | 1.27E-19 |
| MTHFD2 | 3.497348 | 1.07E-20 | 1.28E-19 |
| ALDH3B2 | 3.700925 | 1.09E-20 | 1.30E-19 |
| NME4 | 3.983453 | 1.26E-20 | 1.49E-19 |
| PLA2G4A | 3.40295 | 1.60E-20 | 1.87E-19 |
| RRM2 | 3.543329 | 1.82E-20 | 2.11E-19 |
| NDUFB5 | 4.072252 | 1.86E-20 | 2.14E-19 |
| DDAH1 | 3.568143 | 1.97E-20 | 2.24E-19 |
| UROS | 1.125149 | 2.07E-20 | 2.34E-19 |
| CHST7 | 4.201591 | 2.23E-20 | 2.50E-19 |
| PDE9A | -1.55525 | 2.88E-20 | 3.20E-19 |
| ATP6V0B | 3.406186 | 3.66E-20 | 4.03E-19 |
| TARS | 3.572001 | 3.76E-20 | 4.11E-19 |
| ALPL | 1.815859 | 4.22E-20 | 4.57E-19 |
| HIBADH | 1.874803 | 4.52E-20 | 4.85E-19 |
| NAT2 | -1.1702 | 4.66E-20 | 4.97E-19 |
| ACO2 | -1.58417 | 4.71E-20 | 4.98E-19 |
| GPI | 3.024572 | 5.70E-20 | 5.98E-19 |
| KARS | 2.824887 | 7.81E-20 | 8.12E-19 |
| PLA2G5 | -2.40212 | 9.73E-20 | 1.00E-18 |
| PANK2 | 3.008966 | 1.08E-19 | 1.10E-18 |
| JMJD4 | 3.283594 | 1.52E-19 | 1.54E-18 |
| ME2 | 3.351784 | 1.59E-19 | 1.61E-18 |
| HDHD2 | 3.037253 | 1.68E-19 | 1.68E-18 |
| XDH | 3.000577 | 1.91E-19 | 1.90E-18 |
| ENTPD2 | -1.55675 | 1.97E-19 | 1.94E-18 |
| ARSK | 3.055046 | 2.15E-19 | 2.10E-18 |
| ACOT12 | -1.58214 | 2.54E-19 | 2.46E-18 |
| ALDH3A1 | 2.123515 | 2.69E-19 | 2.59E-18 |
| PRDX1 | 4.335908 | 2.78E-19 | 2.66E-18 |
| SCD | 3.00677 | 3.52E-19 | 3.34E-18 |
| CASP2 | 2.994217 | 3.99E-19 | 3.76E-18 |
| COX7C | 2.759909 | 4.35E-19 | 4.07E-18 |
| ALDH7A1 | 3.215078 | 4.99E-19 | 4.63E-18 |
| MCEE | 3.564028 | 5.11E-19 | 4.71E-18 |
| POLR2I | 2.346738 | 6.09E-19 | 5.57E-18 |
| G6PD | 3.089488 | 7.78E-19 | 7.07E-18 |
| GALC | 3.423264 | 9.35E-19 | 8.45E-18 |
| PCCB | 3.288968 | 1.04E-18 | 9.29E-18 |
| TH | -2.25173 | 1.11E-18 | 9.87E-18 |
| WRNIP1 | 3.364768 | 1.22E-18 | 1.08E-17 |
| STEAP2 | 2.976573 | 1.40E-18 | 1.23E-17 |
| GSTK1 | -1.16524 | 1.47E-18 | 1.28E-17 |
| AMT | 1.836224 | 1.55E-18 | 1.34E-17 |
| CKB | 3.213815 | 2.46E-18 | 2.11E-17 |
| DCTD | 2.270016 | 2.49E-18 | 2.13E-17 |
| OXCT2 | 2.877041 | 2.52E-18 | 2.14E-17 |
| CTPS2 | 3.028168 | 3.28E-18 | 2.77E-17 |
| GLUD1 | 1.733774 | 3.46E-18 | 2.90E-17 |
| CASP4 | 2.999176 | 4.55E-18 | 3.80E-17 |
| PLA2G4D | 3.071793 | 4.82E-18 | 3.99E-17 |
| POLR3B | 1.80035 | 5.17E-18 | 4.25E-17 |
| ASS1 | 2.709833 | 5.71E-18 | 4.67E-17 |
| MCCC2 | 2.677732 | 6.27E-18 | 5.09E-17 |
| ADA | 2.308666 | 6.32E-18 | 5.10E-17 |
| ATP6V1H | 2.985883 | 6.72E-18 | 5.39E-17 |
| NDUFA7 | 3.018892 | 7.47E-18 | 5.96E-17 |
| ST6GALNAC6 | 2.993466 | 7.69E-18 | 6.09E-17 |
| TRMT1 | 2.92561 | 7.96E-18 | 6.27E-17 |
| MGLL | 2.897071 | 8.20E-18 | 6.42E-17 |
| PDE7A | 1.556284 | 8.27E-18 | 6.44E-17 |
| GBA | 3.055133 | 9.68E-18 | 7.49E-17 |
| MOCS1 | 1.765228 | 1.02E-17 | 7.84E-17 |
| AMPD1 | 3.284683 | 1.31E-17 | 1.00E-16 |
| TDO2 | 2.724216 | 1.57E-17 | 1.19E-16 |
| HADHA | 2.485178 | 1.75E-17 | 1.32E-16 |
| ATP5G1 | 3.207706 | 2.74E-17 | 2.06E-16 |
| CBR3 | -2.4651 | 2.98E-17 | 2.22E-16 |
| AHCYL1 | 2.272963 | 3.71E-17 | 2.76E-16 |
| ADHFE1 | 2.145749 | 3.97E-17 | 2.93E-16 |
| PGK1 | 3.711213 | 4.01E-17 | 2.94E-16 |
| LSS | 2.10384 | 4.39E-17 | 3.20E-16 |
| CMAS | 1.967073 | 4.60E-17 | 3.34E-16 |
| ST6GALNAC3 | 2.753326 | 5.05E-17 | 3.65E-16 |
| UMPS | -1.88519 | 5.20E-17 | 3.73E-16 |
| METTL6 | 2.625923 | 6.38E-17 | 4.56E-16 |
| NME1 | 1.828609 | 7.77E-17 | 5.52E-16 |
| PHGDH | 2.118803 | 1.15E-16 | 8.14E-16 |
| LYPLA2 | 2.68967 | 1.20E-16 | 8.45E-16 |
| GPD2 | 1.664872 | 1.23E-16 | 8.63E-16 |
| NAPRT1 | 3.435461 | 1.41E-16 | 9.77E-16 |
| ACPP | 2.92781 | 1.41E-16 | 9.77E-16 |
| ACSL4 | 3.022138 | 1.62E-16 | 1.11E-15 |
| PAICS | 3.499438 | 1.95E-16 | 1.34E-15 |
| RDH12 | 2.834984 | 2.65E-16 | 1.81E-15 |
| GSTM2 | 3.344755 | 2.83E-16 | 1.91E-15 |
| TXNRD2 | 2.635035 | 3.00E-16 | 2.02E-15 |
| TMLHE | 1.307851 | 3.78E-16 | 2.53E-15 |
| POLR3H | 2.428033 | 3.95E-16 | 2.63E-15 |
| GSTO2 | 2.683099 | 6.00E-16 | 3.98E-15 |
| PGM2 | 3.619984 | 6.22E-16 | 4.11E-15 |
| ATP6V1B2 | 3.070295 | 7.16E-16 | 4.70E-15 |
| CDS2 | 2.186825 | 7.32E-16 | 4.78E-15 |
| PMM2 | 2.34176 | 8.80E-16 | 5.72E-15 |
| ABAT | 2.31249 | 8.96E-16 | 5.79E-15 |
| ADCY4 | 2.361125 | 1.01E-15 | 6.47E-15 |
| LIPG | 2.534973 | 1.30E-15 | 8.30E-15 |
| COX6B2 | 2.810294 | 1.37E-15 | 8.75E-15 |
| GAA | 2.344919 | 1.72E-15 | 1.09E-14 |
| ACAD11 | 3.042786 | 1.77E-15 | 1.12E-14 |
| CASP7 | 2.143252 | 1.99E-15 | 1.25E-14 |
| NANS | 2.485595 | 2.20E-15 | 1.37E-14 |
| PDE4D | 1.780825 | 2.43E-15 | 1.51E-14 |
| B4GALT6 | 1.906423 | 2.72E-15 | 1.68E-14 |
| MGST2 | 4.099748 | 3.56E-15 | 2.20E-14 |
| COX7A1 | 2.097646 | 4.10E-15 | 2.51E-14 |
| DNASE2 | 2.614987 | 4.28E-15 | 2.61E-14 |
| PDE8A | 2.005468 | 4.40E-15 | 2.67E-14 |
| CHST11 | -1.80811 | 5.05E-15 | 3.05E-14 |
| PGAM1 | 3.07992 | 5.77E-15 | 3.47E-14 |
| PDE3B | 2.626175 | 6.38E-15 | 3.82E-14 |
| D2HGDH | 2.358828 | 6.65E-15 | 3.96E-14 |
| PRDX2 | 2.337426 | 7.16E-15 | 4.25E-14 |
| NUDT15 | 1.554937 | 9.10E-15 | 5.38E-14 |
| APOA1BP | 2.365383 | 1.06E-14 | 6.26E-14 |
| NDUFA3 | 1.637963 | 1.39E-14 | 8.16E-14 |
| ACADSB | -1.65564 | 1.42E-14 | 8.26E-14 |
| AMY2B | 2.198229 | 1.88E-14 | 1.09E-13 |
| PGLS | 2.555971 | 2.75E-14 | 1.59E-13 |
| AMDHD1 | -1.37313 | 2.87E-14 | 1.65E-13 |
| B4GALT1 | 2.32602 | 3.02E-14 | 1.73E-13 |
| ENTPD7 | 2.425334 | 3.86E-14 | 2.20E-13 |
| DGKQ | 1.751691 | 4.88E-14 | 2.77E-13 |
| IDI2 | -2.1402 | 5.06E-14 | 2.86E-13 |
| CHST10 | 1.947611 | 5.34E-14 | 3.00E-13 |
| ATP5A1 | 1.415563 | 5.67E-14 | 3.18E-13 |
| CHST3 | 2.128702 | 6.02E-14 | 3.36E-13 |
| HMGCR | 1.59454 | 6.08E-14 | 3.38E-13 |
| NDUFB6 | 2.276157 | 7.48E-14 | 4.14E-13 |
| BCAT2 | 2.51527 | 7.69E-14 | 4.24E-13 |
| MCCC1 | 2.414708 | 7.89E-14 | 4.33E-13 |
| GNMT | -2.746 | 9.37E-14 | 5.12E-13 |
| OXCT1 | 2.042521 | 1.05E-13 | 5.70E-13 |
| DHRS1 | 1.316703 | 1.22E-13 | 6.59E-13 |
| POLR2B | 2.054471 | 1.42E-13 | 7.67E-13 |
| GSTM5 | -1.46206 | 1.44E-13 | 7.75E-13 |
| LARS | 2.889121 | 1.55E-13 | 8.29E-13 |
| GNPNAT1 | 2.207013 | 1.58E-13 | 8.44E-13 |
| AKR1C2 | 3.066228 | 1.74E-13 | 9.24E-13 |
| ACOX2 | 1.74824 | 1.81E-13 | 9.58E-13 |
| AK2 | -1.03298 | 1.91E-13 | 1.01E-12 |
| IMPDH2 | 1.710952 | 2.25E-13 | 1.18E-12 |
| ATP6V1G1 | 2.334084 | 3.75E-13 | 1.96E-12 |
| PAH | -1.28789 | 3.76E-13 | 1.96E-12 |
| POLR1C | 1.439482 | 4.19E-13 | 2.17E-12 |
| MMACHC | 2.997969 | 5.01E-13 | 2.59E-12 |
| NDUFS6 | -2.0211 | 5.25E-13 | 2.70E-12 |
| GSTM1 | 2.223895 | 5.39E-13 | 2.76E-12 |
| B4GALT3 | 2.331817 | 5.41E-13 | 2.76E-12 |
| ABHD4 | 1.43294 | 5.88E-13 | 2.99E-12 |
| PNPLA1 | 1.6978 | 7.22E-13 | 3.64E-12 |
| GSTA5 | -2.18363 | 8.22E-13 | 4.13E-12 |
| ECHDC2 | 3.371744 | 8.28E-13 | 4.14E-12 |
| ALDH18A1 | 1.745697 | 9.16E-13 | 4.56E-12 |
| ABHD14A | -2.23289 | 9.31E-13 | 4.62E-12 |
| FUT3 | 2.219818 | 1.13E-12 | 5.61E-12 |
| ACOT8 | 1.82118 | 1.14E-12 | 5.62E-12 |
| ATP6V0C | 2.6011 | 1.23E-12 | 6.01E-12 |
| PEMT | 2.273183 | 1.31E-12 | 6.38E-12 |
| UGT1A8 | -1.38375 | 1.34E-12 | 6.51E-12 |
| LDHB | 1.286209 | 1.43E-12 | 6.92E-12 |
| DLAT | 2.51012 | 1.44E-12 | 6.97E-12 |
| FH | 1.520709 | 1.84E-12 | 8.86E-12 |
| IMPA2 | 1.568683 | 1.97E-12 | 9.44E-12 |
| SDSL | 1.803556 | 2.10E-12 | 1.00E-11 |
| CCBL1 | 1.792031 | 2.64E-12 | 1.26E-11 |
| UQCRFS1 | 2.056099 | 2.69E-12 | 1.28E-11 |
| TBXAS1 | 1.69381 | 3.20E-12 | 1.51E-11 |
| ABHD2 | -2.61922 | 3.39E-12 | 1.59E-11 |
| POLE3 | 1.858958 | 3.46E-12 | 1.62E-11 |
| IDH3B | 2.247643 | 4.36E-12 | 2.02E-11 |
| ARSD | 1.806314 | 4.75E-12 | 2.20E-11 |
| RRM2B | 1.669271 | 5.55E-12 | 2.56E-11 |
| PDHA1 | 1.99133 | 5.64E-12 | 2.59E-11 |
| PNPLA2 | 1.315698 | 6.15E-12 | 2.82E-11 |
| PDE4B | 1.225978 | 6.53E-12 | 2.98E-11 |
| ATP5G2 | 1.058777 | 7.09E-12 | 3.22E-11 |
| GDA | 1.40933 | 7.46E-12 | 3.38E-11 |
| MTO1 | 2.103131 | 8.86E-12 | 4.00E-11 |
| GLB1 | 1.466726 | 9.05E-12 | 4.07E-11 |
| AK3 | 1.855877 | 9.21E-12 | 4.13E-11 |
| CRYL1 | 1.683973 | 9.25E-12 | 4.13E-11 |
| ODC1 | 1.967556 | 1.00E-11 | 4.46E-11 |
| IDH2 | 2.178961 | 1.19E-11 | 5.30E-11 |
| BCKDHA | 2.122079 | 1.24E-11 | 5.49E-11 |
| CDIPT | -2.35942 | 1.33E-11 | 5.84E-11 |
| HEMK1 | 1.667076 | 1.66E-11 | 7.29E-11 |
| ACSM2 | -1.35095 | 1.75E-11 | 7.64E-11 |
| PLA2G12A | 2.164998 | 1.82E-11 | 7.93E-11 |
| NME7 | 1.85154 | 2.05E-11 | 8.90E-11 |
| TXNDC12 | 2.385385 | 2.28E-11 | 9.89E-11 |
| BCAT1 | 1.111 | 2.31E-11 | 9.97E-11 |
| SOD2 | 2.906006 | 2.50E-11 | 1.08E-10 |
| RNASEH1 | 1.947437 | 2.55E-11 | 1.09E-10 |
| PLA2G3 | -1.94157 | 2.80E-11 | 1.20E-10 |
| ABHD5 | 1.625996 | 3.46E-11 | 1.47E-10 |
| AHCY | 1.366397 | 3.55E-11 | 1.51E-10 |
| POLR2A | 1.37925 | 4.01E-11 | 1.70E-10 |
| BPNT1 | 2.070793 | 4.09E-11 | 1.73E-10 |
| PMM1 | 3.041752 | 4.19E-11 | 1.76E-10 |
| COX8A | 1.318522 | 4.54E-11 | 1.90E-10 |
| GPT2 | 2.033901 | 4.66E-11 | 1.95E-10 |
| LIAS | 1.595115 | 5.60E-11 | 2.33E-10 |
| PDK1 | 1.373407 | 5.95E-11 | 2.46E-10 |
| MVD | 1.636743 | 6.00E-11 | 2.48E-10 |
| AADAC | 1.550946 | 6.11E-11 | 2.51E-10 |
| ECH1 | 2.023368 | 6.34E-11 | 2.60E-10 |
| UGT2B11 | -1.90249 | 6.36E-11 | 2.60E-10 |
| NME5 | 1.452795 | 7.89E-11 | 3.22E-10 |
| GLS2 | -1.93961 | 8.37E-11 | 3.40E-10 |
| ENPP4 | -1.13191 | 9.22E-11 | 3.72E-10 |
| NDUFV2 | 1.256211 | 9.24E-11 | 3.72E-10 |
| NMNAT3 | 1.239568 | 9.57E-11 | 3.84E-10 |
| POLD4 | 1.484876 | 9.68E-11 | 3.87E-10 |
| CHST6 | 1.454057 | 1.18E-10 | 4.70E-10 |
| MMAB | 1.847724 | 1.85E-10 | 7.32E-10 |
| ADSS | 1.038116 | 2.10E-10 | 8.25E-10 |
| METTL16 | 1.271726 | 2.19E-10 | 8.61E-10 |
| HARS2 | 1.962924 | 2.30E-10 | 9.01E-10 |
| CS | 2.33057 | 2.43E-10 | 9.47E-10 |
| PPAP2C | -2.00962 | 2.46E-10 | 9.58E-10 |
| GMDS | 3.086038 | 2.74E-10 | 1.06E-09 |
| CHKB | 1.557917 | 2.95E-10 | 1.14E-09 |
| FKBP2 | 1.413792 | 3.24E-10 | 1.25E-09 |
| UROD | 1.123495 | 3.74E-10 | 1.44E-09 |
| FXN | 1.340695 | 4.38E-10 | 1.68E-09 |
| MGMT | 1.346404 | 4.50E-10 | 1.72E-09 |
| PCBD2 | 1.742183 | 4.81E-10 | 1.83E-09 |
| CBR1 | -2.40675 | 4.96E-10 | 1.89E-09 |
| POLM | 1.293846 | 5.47E-10 | 2.07E-09 |
| ACOT2 | -1.75099 | 5.65E-10 | 2.14E-09 |
| CCBL2 | -1.20294 | 7.31E-10 | 2.75E-09 |
| PFKFB3 | 1.517699 | 7.37E-10 | 2.77E-09 |
| PPCDC | -1.2397 | 7.53E-10 | 2.82E-09 |
| METTL5 | 1.385359 | 8.21E-10 | 3.06E-09 |
| RSAD1 | 1.520727 | 9.39E-10 | 3.49E-09 |
| COX4I1 | 2.650444 | 1.14E-09 | 4.21E-09 |
| BLVRB | 1.27931 | 1.17E-09 | 4.32E-09 |
| GPD1 | -1.56062 | 1.25E-09 | 4.57E-09 |
| ATP6V1C2 | 1.612765 | 1.30E-09 | 4.73E-09 |
| HCCS | 2.330218 | 1.30E-09 | 4.73E-09 |
| POLR3G | -1.77844 | 1.33E-09 | 4.84E-09 |
| B4GALT7 | 1.597076 | 1.45E-09 | 5.24E-09 |
| EPHX2 | 1.14499 | 1.49E-09 | 5.38E-09 |
| MOCS3 | 1.107319 | 1.64E-09 | 5.91E-09 |
| TST | 1.330641 | 1.64E-09 | 5.91E-09 |
| COQ7 | 1.690062 | 2.09E-09 | 7.47E-09 |
| ENPP5 | 1.462189 | 2.11E-09 | 7.51E-09 |
| FKBP3 | 1.378028 | 2.13E-09 | 7.57E-09 |
| NDUFS5 | 1.467798 | 2.30E-09 | 8.14E-09 |
| PDE6D | 1.26459 | 2.43E-09 | 8.58E-09 |
| PDHB | 1.223546 | 2.57E-09 | 9.02E-09 |
| CBS | 1.624025 | 2.59E-09 | 9.06E-09 |
| TXNRD1 | 1.334842 | 2.60E-09 | 9.08E-09 |
| MTR | 1.518821 | 2.67E-09 | 9.32E-09 |
| PPA2 | 1.534753 | 2.91E-09 | 1.01E-08 |
| PEO1 | -1.00212 | 3.85E-09 | 1.33E-08 |
| POLRMT | 1.102079 | 4.01E-09 | 1.38E-08 |
| ARSH | -2.38846 | 4.80E-09 | 1.65E-08 |
| CASP1 | 1.568355 | 5.31E-09 | 1.81E-08 |
| DGKE | 1.122641 | 5.55E-09 | 1.89E-08 |
| NDUFC2 | 1.03749 | 5.83E-09 | 1.97E-08 |
| NEU1 | 1.71414 | 6.36E-09 | 2.15E-08 |
| ACADVL | 1.515075 | 6.51E-09 | 2.19E-08 |
| VPS4B | 1.082349 | 6.84E-09 | 2.29E-08 |
| NDUFA1 | 2.172663 | 7.71E-09 | 2.58E-08 |
| GBA3 | 1.167532 | 7.78E-09 | 2.59E-08 |
| MIOX | -2.06903 | 8.07E-09 | 2.68E-08 |
| GLO1 | 1.026061 | 8.19E-09 | 2.72E-08 |
| SQRDL | 1.384378 | 9.58E-09 | 3.16E-08 |
| QARS | 1.877197 | 1.06E-08 | 3.48E-08 |
| NFS1 | 1.012474 | 1.06E-08 | 3.48E-08 |
| IDE | 1.393617 | 1.43E-08 | 4.67E-08 |
| ACP1 | 1.024359 | 1.46E-08 | 4.75E-08 |
| ATP2B3 | -1.66129 | 1.46E-08 | 4.75E-08 |
| ENTPD4 | 1.279044 | 1.62E-08 | 5.24E-08 |
| PDXP | 1.138317 | 1.75E-08 | 5.64E-08 |
| CHST1 | 1.296345 | 2.11E-08 | 6.79E-08 |
| ABHD3 | 1.71902 | 2.23E-08 | 7.14E-08 |
| AMY2A | -1.6905 | 2.28E-08 | 7.30E-08 |
| DDC | 1.448387 | 3.25E-08 | 1.04E-07 |
| CHST4 | -1.64052 | 3.26E-08 | 1.04E-07 |
| FUT6 | -1.4901 | 3.26E-08 | 1.04E-07 |
| ATP6V1F | 1.740199 | 3.32E-08 | 1.05E-07 |
| TDH | 1.29842 | 3.51E-08 | 1.11E-07 |
| CHST13 | 1.731546 | 3.61E-08 | 1.14E-07 |
| COX7B | 1.260314 | 4.05E-08 | 1.27E-07 |
| GUK1 | 2.113849 | 4.43E-08 | 1.39E-07 |
| METTL1 | -1.52067 | 5.27E-08 | 1.63E-07 |
| ACADL | -1.15861 | 5.28E-08 | 1.63E-07 |
| TRMT12 | -1.03361 | 5.52E-08 | 1.71E-07 |
| HSD17B1 | 1.137227 | 5.58E-08 | 1.72E-07 |
| AADAT | -1.33606 | 5.74E-08 | 1.75E-07 |
| DPYD | 1.076026 | 5.75E-08 | 1.75E-07 |
| MECR | 1.001147 | 6.30E-08 | 1.92E-07 |
| MPST | -1.10588 | 6.34E-08 | 1.93E-07 |
| ACLY | 2.166103 | 7.12E-08 | 2.15E-07 |
| DCK | 1.643623 | 7.78E-08 | 2.34E-07 |
| CPS1 | 1.003483 | 1.13E-07 | 3.40E-07 |
| SOD1 | 2.127325 | 1.19E-07 | 3.56E-07 |
| HK3 | -1.4984 | 1.21E-07 | 3.60E-07 |
| ALDH4A1 | 1.633813 | 1.30E-07 | 3.85E-07 |
| PPAPDC1B | 1.358424 | 1.30E-07 | 3.85E-07 |
| MUT | 1.288591 | 1.53E-07 | 4.50E-07 |
| ALDH9A1 | 1.211895 | 1.58E-07 | 4.63E-07 |
| STEAP1 | 1.158898 | 2.10E-07 | 6.09E-07 |
| REXO2 | 1.343267 | 2.10E-07 | 6.09E-07 |
| GLUL | -1.101 | 2.16E-07 | 6.22E-07 |
| FKBP4 | -1.21994 | 2.28E-07 | 6.51E-07 |
| ACOT11 | -1.0371 | 2.31E-07 | 6.58E-07 |
| ETFA | 2.09958 | 2.59E-07 | 7.34E-07 |
| GAL3ST2 | -1.09627 | 2.60E-07 | 7.34E-07 |
| PDK3 | 1.493094 | 2.80E-07 | 7.89E-07 |
| MVK | 1.236486 | 3.55E-07 | 1.00E-06 |
| ATP5G3 | 1.374716 | 4.15E-07 | 1.16E-06 |
| RARS | 1.552818 | 4.36E-07 | 1.21E-06 |
| ATP6V0D2 | 1.326187 | 4.61E-07 | 1.28E-06 |
| MGST1 | 1.033835 | 5.50E-07 | 1.51E-06 |
| MDH2 | 2.587599 | 5.52E-07 | 1.51E-06 |
| PLOD2 | 1.064996 | 6.94E-07 | 1.88E-06 |
| HDC | 1.47446 | 7.28E-07 | 1.96E-06 |
| SPR | -1.30057 | 7.89E-07 | 2.12E-06 |
| COQ6 | 1.209449 | 8.43E-07 | 2.26E-06 |
| NUDT3 | 1.213641 | 1.60E-06 | 4.22E-06 |
| CTH | -1.43331 | 1.72E-06 | 4.52E-06 |
| PRPS1L1 | -1.11492 | 1.83E-06 | 4.79E-06 |
| DBT | 1.014739 | 1.88E-06 | 4.93E-06 |
| PFAS | 1.059077 | 2.45E-06 | 6.40E-06 |
| HK2 | 1.260372 | 2.74E-06 | 7.14E-06 |
| GNPDA2 | -1.34271 | 3.35E-06 | 8.65E-06 |
| PTGS1 | 1.481108 | 3.91E-06 | 1.01E-05 |
| UGT1A6 | 1.007697 | 4.02E-06 | 1.03E-05 |
| EPRS | 1.065896 | 4.73E-06 | 1.21E-05 |
| ST6GALNAC5 | 1.270266 | 4.82E-06 | 1.23E-05 |
| COASY | 1.025329 | 6.49E-06 | 1.64E-05 |
| ACAT1 | 1.008868 | 8.43E-06 | 2.11E-05 |
| PLA2G2A | 1.189154 | 8.48E-06 | 2.12E-05 |
| UGP2 | 1.162696 | 1.46E-05 | 3.56E-05 |
| NMNAT2 | -1.09005 | 1.48E-05 | 3.62E-05 |
| ENPP3 | -1.44574 | 1.51E-05 | 3.67E-05 |
| POLR3D | -1.41284 | 1.99E-05 | 4.79E-05 |
| ACSBG2 | -1.11901 | 3.79E-05 | 8.95E-05 |
| GLYAT | 1.312017 | 3.88E-05 | 9.16E-05 |
| PYCR1 | -1.38534 | 4.50E-05 | 0.000105 |
| FKBP6 | 1.307029 | 4.68E-05 | 0.000109 |
| PDK4 | 1.207021 | 0.000117 | 0.000266 |
| ASAH2 | -1.18997 | 0.000163 | 0.000365 |
| HK1 | 1.291522 | 0.000175 | 0.00039 |
| NUDT4 | -1.006 | 0.000209 | 0.000462 |
| CRAT | -1.45611 | 0.00028 | 0.000602 |
| CASP3 | 1.133387 | 0.00038 | 0.000809 |
| NADSYN1 | -1.15948 | 0.000671 | 0.001376 |
| SCCPDH | 1.01093 | 0.001719 | 0.003278 |
| ENO3 | 1.054838 | 0.006413 | 0.011072 |
